# Practices for running a research-oriented shared cryo-EM facility

**DOI:** 10.3389/fmolb.2022.960940

**Published:** 2022-09-15

**Authors:** Richard M. Walsh, Megan L. Mayer, Christopher H. Sun, Shaun Rawson, Remya Nair, Sarah M. Sterling, Zongli Li

**Affiliations:** ^1^ Harvard Cryo-EM Center for Structural Biology, Blavatnik Institute, Harvard Medical School, Boston, MA, United States; ^2^ Department of Biological Chemistry and Molecular Pharmacology, Blavatnik Institute, Harvard Medical School, Boston, MA, United States; ^3^ Howard Hughes Medical Institute, Harvard Medical School, Boston, MA, United States

**Keywords:** cryo-EM, shared facility, user training, sample optimization, on-the-fly pre-processing

## Abstract

The Harvard Cryo-Electron Microscopy Center for Structural Biology, which was formed as a consortium between Harvard Medical School, Boston Children’s Hospital, Dana-Farber Cancer Institute, and Massachusetts General Hospital, serves both academic and commercial users in the greater Harvard community. The facility strives to optimize research productivity while training users to become expert electron microscopists. These two tasks may be at odds and require careful balance to keep research projects moving forward while still allowing trainees to develop independence and expertise. This article presents the model developed at Harvard Medical School for running a research-oriented cryo-EM facility. Being a research-oriented facility begins with training in cryo-sample preparation on a trainee’s own sample, ideally producing grids that can be screened and optimized on the Talos Arctica via multiple established pipelines. The first option, staff assisted screening, requires no user experience and a staff member provides instant feedback about the suitability of the sample for cryo-EM investigation and discusses potential strategies for sample optimization. Another option, rapid access, allows users short sessions to screen samples and introductory training for basic microscope operation. Once a sample reaches the stage where data collection is warranted, new users are trained on setting up data collection for themselves on either the Talos Arctica or Titan Krios microscope until independence is established. By providing incremental training and screening pipelines, the bottleneck of sample preparation can be overcome in parallel with developing skills as an electron microscopist. This approach allows for the development of expertise without hindering breakthroughs in key research areas.

## Introduction

Cryo-electron microscopy (cryo-EM) has emerged as a major tool for structural biology in last decade (Kuhlbrandt, 2014; Cheng et al., 2015), thanks to advances in direct electron detectors (Faruqi and McMullan, 2011; Milazzo et al., 2011; Brilot et al., 2012; Li et al., 2013), electron microscope optics (Thermo Fisher Scientific, Inc.; Jeol Ltd.) and image processing (Grigorieff, 2007; Tang et al., 2007; Scheres, 2012; Punjani et al., 2017; Grant et al., 2018). Cryo-EM can be used to investigate a wide range of biological specimens preserved close to physiological conditions, providing near-atomic details of purified macromolecules or protein complexes (single particle analysis, SPA) (Liao at al., 2013; Lu et al., 2014; Liang et al., 2015; Duan et al., 2019) and less-detailed views of macromolecules in a native cellular environment (cryo electron tomography, cryo-ET) (Dierksen et al., 1995; Förster et al., 2005; Villa et al., 2013; Oikonomou et al., 2016; Ke et al., 2020; Erdmann et al., 2021; Ni et al., 2021; Guo et al., 2022). As more researchers utilize cryo-EM for structural determination, access to high-end cryo-EM instrumentation becomes a bottleneck due to the high costs for instrument use and lack of cryo-EM expertise. To address this challenge, government agencies across the globe have invested heavily to establish large national cryo-EM centers (Zimanyi et al., 2022), many of which provide academic users free access for data collection and user training. In addition, local, smaller scale cryo-EM centers have been established through collaborations among institutions, or by universities with support from various funding sources (https://pncc.labworks.org/team/cryoem-service-centers-working-list). Large government-funded cryo-EM centers have the resources to hire multidisciplinary experts and tackle sophisticated problems, such as sample preparation techniques, specialized software development, and machine learning exploration in data collection, particle picking or data analysis. Local cryo-EM centers have the flexibility to be project-focused with a smaller scale of operation and close connection with local research laboratories. A smaller user base enables staff to be familiar with individual projects and their progress, allowing for helpful suggestions and advice to users in achieving their scientific goals. Most local cryo-EM centers have standard protocols for data collection and user training; however, the formal training process often needs to be tailored to match a specific user’s strengths and weaknesses. Here we present the daily practices of the Harvard Cryo-EM Center for Structural Biology (HC^2^EM), to fulfill our mission in helping researchers to advance their scientific investigation and training the next generation of cryo-EM experts.

## Daily operation at HC^2^EM

**FIGURE 1:**
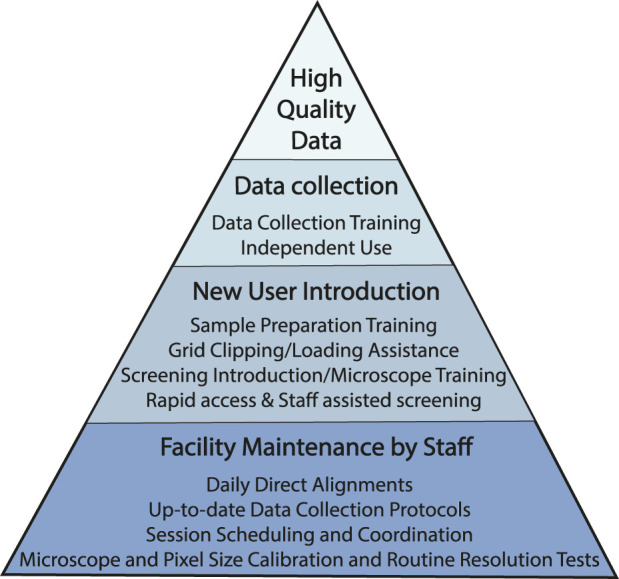
User Success Pyramid. Pyramid of steps taken to promote high quality data collections for all users. At the base of the pyramid are routine facility and microscope maintenance tasks to ensure that microscope operation is of the highest standard. New users are introduced to best practices so that they are familiar and comfortable with baseline data collection workflow; users gradually develop the skills necessary to collect data independently. All necessary microscope and imaging parameters for all imaging sessions are documented in a standardized datasheet which is emailed to the user at the end of each session (see Supplementary Appendix S1).

At HC^2^EM, we strive to optimize usage efficiency and minimize instrument downtime via the implementation of standardized protocols, sufficient staff-to-trainee ratio to provide adequate training and support, and reserving routine maintenance tasks for experienced staff scientists (Figure 1. User Success Pyramid). Single particle analysis of purified protein is now a routine procedure and automated collection software packages follow the same workflow: collect a low magnification overview of the entire grid (LMM), acquire multiple medium magnification maps (MMMs) at eucentric height, select data acquisition and autofocusing targets on the MMMs, and run automated data collection on the selected data acquisition targets. Of the three main automated software packages in use (EPU [Thermo Fisher Scientific, Inc.], SerialEM (Mastronarde, 2005) and Leginon (Suloway et al., 2005)), our facility has evolved to prefer SerialEM for its fast, automated, and customizable scripts that are freely available from other facilities (https://sphinx-emdocs.readthedocs.io/en/latest/index.html; Nexperion, https://serialemscripts.nexperion.net).

Microscope-level direct alignments (pivot points, aperture alignments, astigmatism, condenser alignments, rotation center, and coma-free alignments) are performed by staff daily to maintain beam stability and ensure data collection quality. These microscope level alignments are gradually introduced to experienced researchers -- we do not expect new users to master them after a few short training sessions spaced out over several weeks. In addition to the daily direct alignments, SerialEM and Thermo Fisher Scientific’s TEM-UI allow staff to save and revert to previous stable settings when the beam is badly misaligned due to user or service error, or changed for experimental purposes. The ability to test, save and revert to stable settings allows staff to maintain baseline microscope imaging conditions which are loaded at the start of each new session.

All data collection protocols at HC^2^EM are tested for reproducibility and documented on our website (https://cryoEM.hms.harvard.edu). Each protocol is tailored to a range of user experience levels, and reviews necessary steps to perform the desired task with high quality results, i.e., how to collect LMM and MMMs, how to align between magnifications, and how to pick data collection points and set up dose parameters. To ensure the written directions are communicated clearly, staff go over each step of the workflow with every new user until familiarity is developed by performing the tasks independently. At the end of each session, relevant microscope setup details and imaging parameters are recorded in a microscope datasheet form (see Datasheet in Supplementary Appendix S1) which is shared with each user electronically. This allows researchers to maintain all the appropriate documentation for their experiments, even without previous cryoEM experience.

While we have protocols in place that allow new users to collect high quality data during their first few sessions, experienced users such as advanced cryo-EM/ET investigators and/or industrial users who are interested in more experimental work, are not expected to follow the protocols line-for-line. We have developed an independent user guide for evenings and weekends which outlines basic alignments as well as troubleshooting steps for potential errors in the absence of staff. During these independent and/or experimental sessions, typical microscope and SerialEM settings may be changed as long as they are reverted to previous stable versions at the end of the session.

Lastly, staff visually inspect all grids to ensure they are clipped properly before loading which eliminates downtime due to autoloader and beam path blockage errors. Users are expected to deliver their grids at least three business days in advance of their session, giving staff ample time to organize grid loading logistics. To facilitate the travel of grids through the facility, a grid inventory must be submitted for all sessions (see Grid Inventory in Supplementary Appendix S2) which requires basic information about the planned experiment and communicates the exact location of each grid to be loaded. The grid inventory tracks the path of a grid from delivered grid buttons to the microscope cassette, then to an autogrid storage box and ultimately back to the user without needing them to be present during clipping or loading.

## Consultation

With a staff cohort of varying backgrounds and experience, users can access different perspectives for their projects, and there are multiple ways to gain access to staff members. We have a centralized email address which goes to all staff members, allowing staff to discuss incoming questions collaboratively. This email is commonly used by external and commercial users to contact the facility. We also have a consultation form in our scheduling platform. This second pathway to staff is a conduit for consortium or internal users to get one-on-one assistance for their projects. Usually, there is a discussion among the staff about who will respond, and that staff member is given freedom to assist as they see appropriate. From this form and email communications, there have been in-person meetings to tour the facility, virtual meetings to review images together, formal collaborations and most often, the scheduling of a training session to move the user’s project forward. Consultation with staff members is not limited to cryo-EM techniques of plunging samples or data collection. We discuss protein purification methodology and sample additives such as detergent or glycerol, biochemical sample characterization, and grid type and treatment such as graphene oxide or thin carbon films. We keep a list of recommended tools and resources for users who are new to cryo-EM. With a dedicated cryo-EM computational specialist, we offer training and consultation on data processing and plan to move toward assistance with figure preparation for manuscripts.

## Access

**FIGURE 2:**
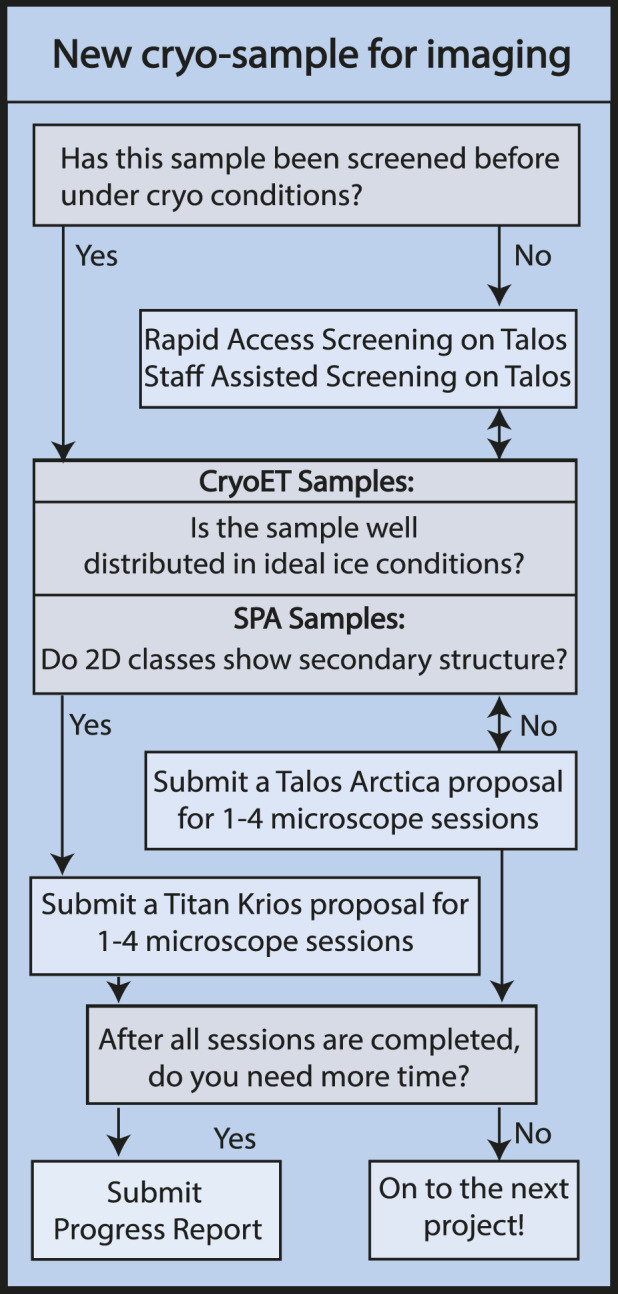
Proposal Flowchart. Flowchart illustrating the steps of obtaining microscope access for new academic research projects via HC^2^EM microscope proposal process. Industry users gain facility access via consultation with staff, pending availability.

Access to instrumentation follows a non-proposal pipeline for short screening sessions (described in Sample optimization) and a proposal-based system for longer data collection sessions (Figure 2. Proposal Flowchart). The proposal system is intended to promote efficient use and appropriate allocation of instrumentation. The consortium members at the HC^2^EM have differing allocations of time and each institution is responsible for determining how their allocation is distributed amongst their respective users. Each institution has a review committee comprising structural biology faculty that regularly utilize the HC^2^EM. For a proposal, the user must describe their project, including details about the specimen, a brief background, and the biological relevance of the project. For specimen screening on the Talos Arctica, the proposal must include biochemical data and ideally negative stain images or 2D classes. For data collection, the proposal must include cryo-EM images or 2D classes showing secondary structure with a description of the data collection and processing scheme. The proposals are curated by the facility staff and sent to the appropriate institutional review committee. The review committee decides the number of sessions each project is permitted, with a maximum of four sessions possible. The review committee’s decision is sent to the facility staff who then set up trackable projects in the scheduling platform so the project status is readily accessible by the user and facility staff.

Talos Arctica sessions are usually defined in 24-h increments. Titan Krios sessions are at least 24-h in length with additional days scheduled as needed for the project. Once the user has completed their allotted number of sessions, they may submit a progress report to request additional sessions on a given microscope. The progress report may also be used to move a project from the Talos Arctica to the Titan Krios. Progress reports require the user to describe why more time is needed, including pitfalls they have experienced. Progress reports for the Titan Krios require comparable 2D classes or 3D reconstructions while the Talos Arctica progress report is tailored to only need further biochemical characterization. Once submitted, the progress reports undergo the same review process by the institutional committees as described for the proposals. The user is notified and the number of sessions available for a given project is updated in the scheduling platform.

For academic users outside of the consortium, proposals and progress reports are reviewed by a combination of consortium member faculty who also participate in the review process for their respective institution. External academic users are given full access to the facility and scheduling platform for their sessions.

## Sample optimization

**FIGURE 3:**
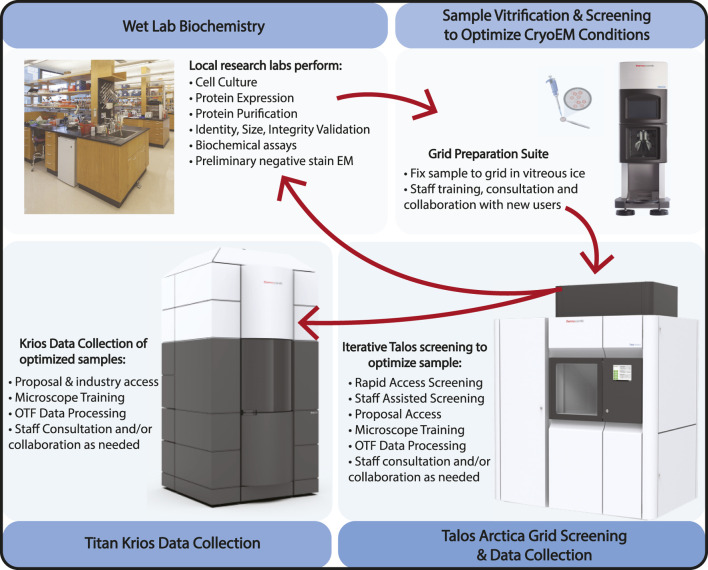
Sample Optimization Flowchart. The cryo-EM sample optimization process at HC^2^EM starts with biochemical identification and evaluation in neighboring wet labs, iterative grid freezing and image screening on the Talos Arctica to refine and optimize cryo conditions, and final high-resolution data collection on either the Talos Arctica or Titan Krios. For academic users, the Talos microscope can be used for general data collection but also has to become simply proposal-free screening routes that allow users to generate data required to submit a Titan Krios proposal. Vitrobot, Talos Arctica, and Titan Krios are products of Thermo Fisher Scientific, Inc. The pipette and grid image were created by Biorender.com. The biochemical lab photograph was taken of the Department of Microbiology and Immunobiology of Harvard Medical School (www.hms.harvard.edu).

The quality of structural information obtained through cryo-EM is highly dependent on specimen preparation. Cryo-EM leverages a process called vitrification, which involves plunging an EM grid with a thin layer of biological specimen suspended over holey carbon or gold foil (or other substrate) into liquid ethane surrounded by liquid nitrogen. The preparation of cryo-EM samples is challenging because freezing conditions vary for different samples, and the important properties of the cryo-EM sample, such as the ice thickness and particle distribution, are hard to control and standardize in a protocol. Cryo-EM specimen preparation is considered the current bottleneck in the field, and many groups are working on improving this process [see reviews by Weissenberger et al. (2021); Han et al. (2022)]. In our facility, users are trained on the Vitrobot [Thermo Fisher Scientific, Inc.], using their own samples, with plunging parameters based on staff experience or the parameters of others in our EM community working on similar samples. Staff familiarity with individual projects and their progress allows the introduction of new trainees to experienced users who can best expedite overcoming their specific sample preparation hurdles (Figure 3. Sample Optimization Flowchart).

To facilitate rapid sample optimization, we have two weekly proposal-free pipelines: staff assisted screening (SAS) and rapid access sessions (RA). The first pipeline, staff assisted screening, only requires that users provide cryo-samples. In fact, the first requested SAS often utilizes the samples produced during the initial Vitrobot training for cryo-sample preparation. During the screening session, a staff member will collect LMMs of each grid and high magnification screening images to assess particle concentration and ice quality. Users are required to be present during the screening to learn about the process of sample evaluation: how to judge ice thickness from the appearance of grid squares at low and medium magnifications and assessing the effect of ice thickness on particle distributions. During the screening process, staff members discuss ways with the user to optimize sample preparation and provide feedback on general sample quality. Morning timeslots are for grid screening only while the final afternoon timeslot has the option for overnight data collection set up by a staff member. This overnight collection is paired with on-the-fly pre-processing (see more detail in the following section) to provide initial feedback on the sample/data evaluation without needing to understand all the steps of data processing.

The second pipeline, RA, is for users who only want to use the microscope for a fraction of the day for screening and collecting small datasets. The morning session is limited to 4 h of use while the afternoon session has the option to collect data overnight. LMMs of the submitted samples are collected by staff before the user begins their session, providing a global view of general ice quality as a starting point for the screening process. Users are given introductory training to basic microscope operation, teaching them how to collect data manually using SerialEM. These basic steps: 1) go to a position (XY), 2) find the Z height, 3) center on a sample hole, 4) autofocus, and 5) take a high magnification image, can be iterated for duration of the screening. This allows users to draw conclusions about their samples at the single micrograph level: particle distribution, particle density and compositional homogeneity, whether there is a strong preferred orientation and the effect of differing ice thicknesses on these and other properties. If a sample looks promising by these metrics, users are guided through the process of setting up an automated data collection.

## On-the-fly pre-processing

**FIGURE 4:**
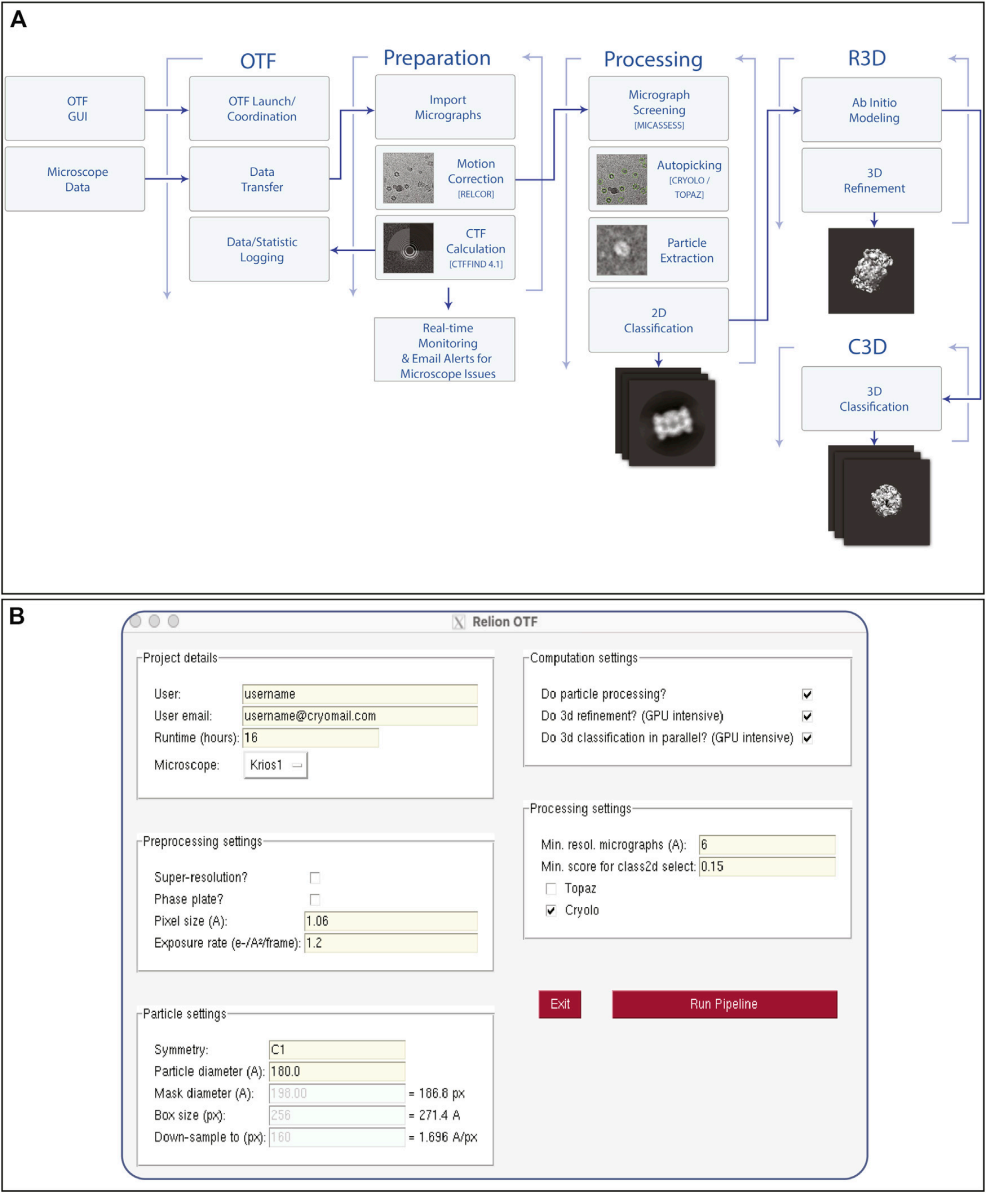
On-The-Fly Pre-Processing Pipeline. Once data collection begins on any microscope, the user will launch an on-the-fly (OTF) pre-processing Scheme **(A)** via a graphical user interface **(B)**. once launched, various processing steps are initiated in real time such as motion correction, CTF estimation, as well as downstream particle picking, classification and refinement steps. Email alerts are sent to both the user and facility staff when resolution quality does not meet a certain threshold, or if data collection stops due to unexpected microscope errors.

Due to the high cost of purchasing, maintaining and running high-end EM equipment (such as a Titan Krios with energy filter and phase plate, equipped with a direct electron detector camera), microscope time is still very expensive (estimated at ∼£3,000 per day) (Passmore and Russo, 2016). Microscope time is often not used efficiently as the quality and value of a data collection can only be determined long after the collection has been completed. Additionally, despite microscope operation and collection of high-resolution data becoming more routine (Carragher et al., 2000; Mastronarde, 2005; Suloway et al., 2005; Schorb et al., 2019) the instruments are still sensitive to a range of factors, from grid contaminants to room temperature, and they can often fail during automated collection. Due to these issues, there is considerable benefit from the introduction of the same kinds of automation which are now routine within the x-ray community, particularly within synchrotron sources. We have implemented a pre-processing pipeline based around the relion_it.py script (Kimanius et al., 2021). As shown in Figure 4A, this performs all the initial processing steps (motion correction, CTF estimation, particle picking, in addition to 2D classification and initial 3D model generation, 3D refinement and 3D classification) in parallel with additional logging and monitoring systems which allow for rapid identification of issues in the data collection since data quality is assessed in real time. These automated processing jobs allow novice users to begin to interpret their results without requiring a deep understanding of all processing steps.

The pipeline can be initiated via a graphical user interface (Figure 4B), which has a minimal number of parameters required from the user. Once the user has initiated the pipeline, file transfer runs automatically in the background, first copying, and later removing the raw images from the microscope/camera data storage system. The computational pipeline is then triggered, leveraging the power of the RELION scheme framework to carry out the processing steps, alongside the monitoring functions. This process continues until the user-specified end of the data collection. All the software packages used within the pipeline are available via SBGrid, which enables easy updating of all components and software. The core of the workflow uses the RELION software package for many tasks (Fernandez-Leiro and Scheres, 2017). This is the most used EM package based on EMDB deposition statistics (in 2020, ∼50% (1,739 of the 3,492) annotated structures were processed within RELION), giving users the data in a familiar format, thus minimizing the training required. Some of the operations, including CTF estimation (Rohou and Grigorieff, 2015) and particle picking within topaz (Bepler et al., 2019) or crYOLO (Wagner et al., 2019), only use RELION as a wrapper to keep data types consistent throughout.

## Facility status

**FIGURE 5:**
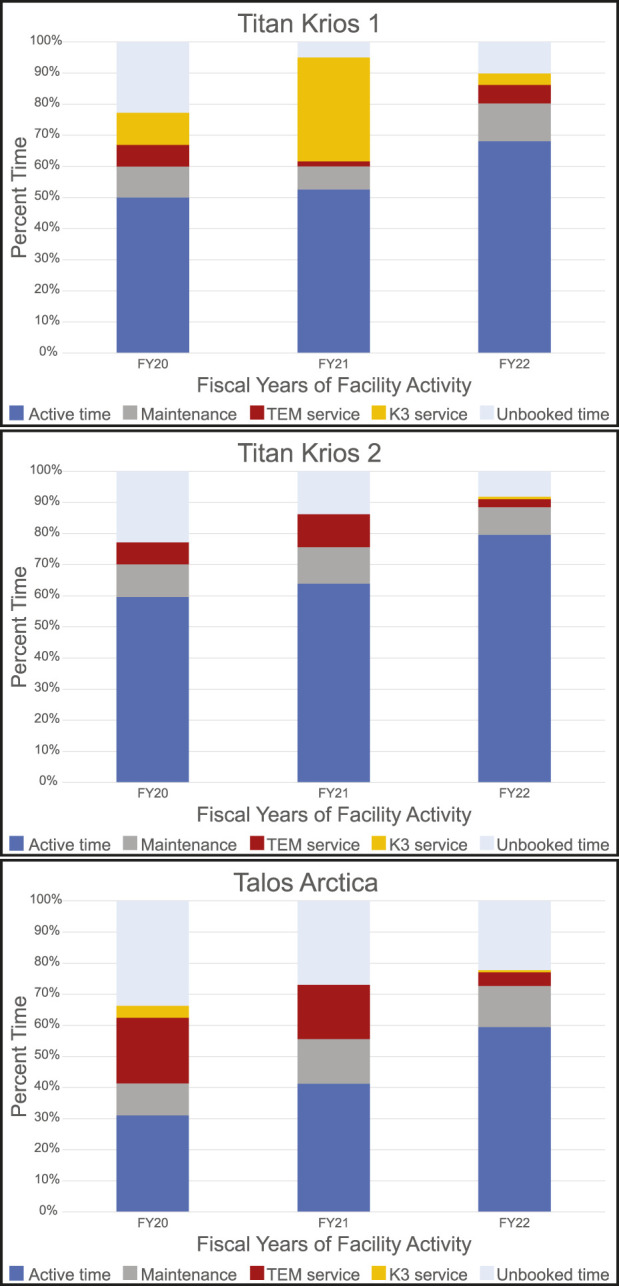
Microscope Utilization. Microscope utilization from July 2019 through June 2022 broken down by fiscal year (July to June) for each microscope. Active time is user sessions and staff collaborative work. Maintenance includes cryo-cycles and preventative maintenance by staff and service engineers for all equipment. Service time required a Thermo Fisher Scientific or Gatan service engineer to intervene as the microscope was not usable.

While the methods outlined above have changed over time, they have served as the current practice at the HC^2^EM since March 2021, the latest addition being the RA sessions. Our facility opened to users in February 2019 after several months of uncharged use. This time was to introduce users to the facility and to allow staff to develop internal workflows and protocols. Approximately 200 users have been trained on the Vitrobot or have used the microscopes. To date, 221 project proposals have been submitted with 164 currently active between the three microscopes. Figure 5 provides a snapshot of the use of the three microscopes for three fiscal years (July 2019—June 2022). The microscopes have been utilized more each year for data collection and screening while the idle time has decreased. The introduction of SAS and RA sessions increased booked time on the Talos Arctica. We average 11 RA sessions and 10 SAS sessions per month with about 8 of those sessions leading to overnight data collection. We have experienced the same service hurdles that many microscope owners face; we have Thermo Fischer Scientific microscopes equipped with Gatan K3 camera systems that require periodic service. The downtime noted by TEM service or K3 service has not been for any user or staff misuse of the instrumentation (e.g., improperly clipped grids). Our downtime has been for equipment failures, for example, a broken spring in the autoloader, a loose cassette gripper, camera power supply failure, or chilled water issues. Once our issues are resolved, the staff takes additional time to confirm that the microscope and camera are still performing optimally often by collecting a dataset of apoferritin.

Another metric we have tracked at the HC^2^EM is the publication record. To date, 50 publications have acknowledged our facility and are listed on our website (https://cryoEM.hms.harvard.edu). This work represents results from each of the HC^2^EM founding institutions, other external academic institutions, and commercial users.

## Conclusion

Unique advantages of cryo-EM, over x-ray crystallography and NMR, paired with recent advances in both instrumentation and software development in last decade, has led to year over year increases in structural determination using cryo-EM (https://www.rcsb.org/stats/all-released-structures). Many national and regional cryo-EM centers have been established through various resources to meet this fast-growing demand for access to high-end cryo-EM and high-performance computing. However, running these facilities smoothly and efficiently is not trivial. At the Harvard Cryo-EM Center for Structural Biology, we strive to prioritize research progress while training users to become expert electron microscopists. To accomplish this goal, we utilize standardized protocols to provide incremental training and have sufficient staff to provide constant support: assisting, advising and encouraging users and projects forward. Our smaller user base allows us to be familiar with individual users and their projects, and we can tailor training based on current and desired skill level. We work closely with member institutions to allocate the microscope time to each research lab and provide consultations to users, helping them to address their specific problems from start through the completion of their projects. Optimizing preparation of a cryo-EM specimen can be challenging when starting a new biological project, especially for researchers new to the field. By pairing Vitrobot training with two approaches to cryo-EM screening—SAS and RA, with help from our experienced staff scientists—users can get quick feedback about the quality of their cryo-EM specimen and make appropriate adjustments when preparing more samples. This has greatly expedited the screening process, increasing trainee productivity and improving the efficiency of microscope usage. Finally, the on-the-fly pre-processing gives real-time feedback for data collection and reduces lost time while collecting data when samples are not optimal. In addition, our staff members can collaborate with individual labs or researchers on various projects, helping them with sample preparation, screening, data collection or processing. We have observed these practices to be a positive methodology for our 3 years of operation and hope they are useful for other cryo-EM facilities.

## Data Availability

The original contributions presented in the study are included in the article/Supplementary Material, further inquiries can be directed to the corresponding author.

## References

[B1] Bepler T. Morin A. Rapp M. Brasch J. Shapiro L. Noble A. J. (2019). Positive-unlabeled convolutional neural networks for particle picking in cryo-electron micrographs. Nat. Methods 16 (11), 1153–1160. 10.1038/s41592-019-0575-8 31591578 PMC6858545

[B2] Brilot A. F. Chen J. Z. Cheng A. Pan J. Harrison S. C. Potter C. S. (2012). Beam-induced motion of vitrified specimen on holey carbon film. J. Struct. Biol. 177 (3), 630–637. 10.1016/j.jsb.2012.02.003 22366277 PMC3322646

[B3] Carragher B. Kisseberth N. Kriegman D. Milligan R. A. Potter C. S. Pulokas J. (2000). Leginon: An automated system for acquisition of images from vitreous ice specimens. J. Struct. Biol. 132 (1), 33–45. 10.1006/jsbi.2000.4314 11121305

[B4] Cheng Y. Grigorieff N. Penczek P. A. Walz T. (2015). A primer to single-particle cryo-electron microscopy. Cell 161 (3), 438–449. 10.1016/j.cell.2015.03.050 25910204 PMC4409659

[B5] Dierksen K. Typke D. Hegerl R. Walz J. Sackmann E. Baumeister W. (1995). Three-dimensional structure of lipid vesicles embedded in vitreous ice and investigated by automated electron tomography. Biophys. J. 68 (4), 1416–1422. 10.1016/S0006-3495(95)80314-0 7787027 PMC1282036

[B6] Duan J. Li J. Chen G. L. Ge Y. Liu J. Xie K. (2019). Cryo-EM structure of TRPC5 at 2.8-Å resolution reveals unique and conserved structural elements essential for channel function. Sci. Adv. 5 (7), eaaw7935. 10.1126/sciadv.aaw7935 31355338 PMC6656536

[B7] Erdmann P. S. Hou Z. Klumpe S. Khavnekar S. Beck F. Wilfling F. (2021). *In situ* cryo-electron tomography reveals gradient organization of ribosome biogenesis in intact nucleoli. Nat. Commun. 12 (1), 5364. 10.1038/s41467-021-25413-w 34508074 PMC8433212

[B8] Faruqi A. R. McMullan G. (2011). Electronic detectors for electron microscopy. Q. Rev. Biophys. 44 (3), 357–390. 10.1017/S0033583511000035 21524337

[B9] Fernandez-Leiro D. Scheres S. H. W. (2017). A pipeline approach to single-particle processing in RELION. Acta Crystallogr. D. Struct. Biol. 73 (6), 496–502. 10.1107/S2059798316019276 28580911 PMC5458491

[B10] Förster F. Medalia O. Zauberman N. Baumeister W. Fass D. (2005). Retrovirus envelope protein complex structure *in situ* studied by cryo-electron tomography. Proc. Natl. Acad. Sci. U. S. A. 102 (13), 4729–4734. 10.1073/pnas.0409178102 15774580 PMC555690

[B11] Grant T. Rohou A. Grigorieff N. (2018). cisTEM, user-friendly software for single-particle image processing. eLife 7, e35383. 10.7554/eLife.35383 29513216 PMC5854467

[B12] Grigorieff N. (2007). Frealign: High-resolution refinement of single particle structures. J. Struct. Biol. 157 (1), 117–125. 10.1016/j.jsb.2006.05.004 16828314

[B13] Guo S. Xu H. Chang Y. Motaleb M. A. Liu J. (2022). Flil ring enhances the function of periplasmic flagella. Proc. Natl. Acad. Sci. U. S. A. 119 (11), e2117245119. 10.1073/pnas.2117245119 35254893 PMC8931381

[B14] Han B. G. Armstrong M. Fletcher D. A. Glaeser R. M. (2022). Perspective: Biochemical and physical constraints associated with preparing thin specimens for single-particle cryo-EM. Front. Mol. Biosci. 9, 864829. 10.3389/fmolb.2022.864829 35573724 PMC9100935

[B15] Ke Z. Oton J. Qu K. Cortese M. Zila V. McKeane L. (2020). Structures and distributions of SARS-CoV-2 spike proteins on intact virions. Nature 588 (7838), 498–502. 10.1038/s41586-020-2665-2 32805734 PMC7116492

[B16] Kimanius D. Dong L. Sharov G. Nakane T. Scheres S. H. W. (2021). New tools for automated cryo-EM single-particle analysis in RELION-4.0. Biochem. J. 478 (24), 4169–4185. 10.1042/BCJ20210708 34783343 PMC8786306

[B17] Kuhlbrandt W. (2014). Biochemistry. The resolution revolution. Science 343 (6178), 1443–1444. 10.1126/science.1251652 24675944

[B18] Li X. Mooney P. Zheng S. Booth C. Braunfeld M. B. Gubbens S. (2013). Electron counting and beam-induced motion correction enable near-atomic-resolution single-particle cryo-EM. Nat. Methods 10 (6), 584–590. 10.1038/nmeth.2472 23644547 PMC3684049

[B19] Liang B. Li Z. Jenni S. Rahmeh A. A. Morin B. M. Grant T. (2015). Structure of the L protein of vesicular stomatitis virus from electron cryomicroscopy. Cell 162 (2), 314–327. 10.1016/j.cell.2015.06.018 26144317 PMC4557768

[B20] Liao M. Cao E. Julius D. Cheng Y. (2013). Structure of the TRPV1 ion channel determined by electron cryo-microscopy. Nature 504 (7478), 107–112. 10.1038/nature12822 24305160 PMC4078027

[B21] Lu P. Bai X. C. Ma D. Xie T. Yan C. Sun L. (2014). Three-dimensional structure of human γ-secretase. Nature 512 (7513), 166–170. 10.1038/nature13567 25043039 PMC4134323

[B22] Mastronarde D. N. (2005). Automated electron microscope tomography using robust prediction of specimen movements. J. Struct. Biol. 152 (1), 36–51. 10.1016/j.jsb.2005.07.007 16182563

[B23] Milazzo A.-C. Cheng A. Moeller A. Lyumkis D. Jacovetty E. Polukas J. (2011). Initial evaluation of a direct detection device detector for single particle cryo-electron microscopy. J. Struct. Biol. 176 (3), 404–408. 10.1016/j.jsb.2011.09.002 21933715 PMC3210420

[B24] Ni T. Zhu Y. Yang Z. Xu C. Chaban Y. Nesterova T. (2021). Structure of native HIV-1 cores and their interactions with IP6 and CypA. Sci. Adv. 7 (47), eabj5715. 10.1126/sciadv.abj5715 34797722 PMC8604400

[B25] Oikonomou C. M. Chang Y. Jensen G. J. (2016). A new view into prokaryotic cell biology from electron cryotomography. Nat. Rev. Microbiol. 14 (4), 205–220. 10.1038/nrmicro.2016.7 26923112 PMC5551487

[B26] Passmore L. A. Russo C. J. (2016). Specimen preparation for high-resolution cryo-EM. Methods Enzymol. 579, 51–86. 10.1016/bs.mie.2016.04.011 27572723 PMC5140023

[B27] Punjani A. Rubinstein J. L. Fleet D. J. Brubaker M. A. (2017). cryoSPARC: algorithms for rapid unsupervised cryo-EM structure determination. Nat. Methods 14 (3), 290–296. 10.1038/nmeth.4169 28165473

[B28] Rohou A. Grigorieff N. (2015). CTFFIND4: Fast and accurate defocus estimation from electron micrographs. J. Struct. Biol. 192 (2), 216–221. 10.1016/j.jsb.2015.08.008 26278980 PMC6760662

[B29] Scheres S. H. W. (2012). Relion: Implementation of a bayesian approach to cryo-EM structure determination. J. Struct. Biol. 180 (3), 519–530. 10.1016/j.jsb.2012.09.006 23000701 PMC3690530

[B30] Schorb M. Haberbosch I. Hagen W. J. H. Schwab Y. Mastronarde D. N. (2019). Software tools for automated transmission electron microscopy. Nat. Methods 16 (6), 471–477. 10.1038/s41592-019-0396-9 31086343 PMC7000238

[B31] Suloway C. Pulokas J. Fellmann D. Cheng A. Guerra F. Quispe J. (2005). Automated molecular microscopy: The new Leginon system. J. Struct. Biol. 151 (1), 41–60. 10.1016/j.jsb.2005.03.010 15890530

[B32] Tang G. Peng L. Baldwin P. R. Mann D. S. Jiang W. Rees I. (2007). EMAN2: An extensible image processing suite for electron microscopy. J. Struct. Biol. 157 (1), 38–46. 10.1016/j.jsb.2006.05.009 16859925

[B33] Villa E. Schaffer M. Plitzko J. M. Baumeister W. (2013). Opening windows into the cell: Focused-ion-beam milling for cryo-electron tomography. Curr. Opin. Struct. Biol. 23 (5), 771–777. 10.1016/j.sbi.2013.08.006 24090931

[B34] Wagner T. Merino F. Stabrin M. Moriya T. Antoni C. Apelbaum A. (2019). SPHIRE-crYOLO is a fast and accurate fully automated particle picker for cryo-EM. Commun. Biol. 2, 218. 10.1038/s42003-019-0437-z 31240256 PMC6584505

[B35] Weissenberger G. Henderikx R. J. M. Peters P. J. (2021). Understanding the invisible hands of sample preparation for cryo-EM. Nat. Methods 18 (5), 463–471. 10.1038/s41592-021-01130-6 33963356

[B36] Zimanyi C. M. Kopylov M. Potter C. S. Carragher B. Eng E. T. (2022). Broadening access to cryoEM through centralized facilities. Trends biochem. Sci. 47 (2), 106–116. 10.1016/j.tibs.2021.10.007 34823974 PMC8760164

