# Supplementary material for: Practices for running a research-oriented shared cryo-EM facility
Source: Front Mol Biosci. 2022 Sep 15;9:960940. doi: 10.3389/fmolb.2022.960940 (PMC9521047; doi:10.3389/fmolb.2022.960940)
Supplement: Supplementary file 1 [file Presentation1.pdf]

Appendix I. **Datasheet.** Datasheet to record all necessary microscope and imaging parameters.

HARVARD
MEDICAL SCHOOL

Data Collection Parameters

**General**	
User	
Date	
Detector mode	
Pixel size (Å)	
Nominal magnification	
Beam setting/mode (μ or n)	
Spot size	
Illuminated area	
**Dose**	
Dose per physical pixel per second	
Dose per $Å^2$ per second	
Exposure time (s)	
Total dose ($e/Å^2$)	
Number of frames	
Dose per frame ($e/Å^2$)	
**Other**	
Desired defocus range	
C2 aperture size (micron)	
Objective aperture size (micron)	
Energy filter slit width (eV)	
Tilt angle	
Phase plate positions	
SerialEM version	
**Processing**	
Particle diameter (Å)	
Number of shots per hole	
Number of holes per image shift	
Script run time (hours)	
Script start time	

Appendix II. **Grid Inventory.** Grid inventory to track user grids before and after microscope sessions.

User/Lab:	Session Date:	Project ID:
Pixel size:	BSL 2? No ☐ Yes ☐ →If Yes, disposal method:	
Tube name:	Special requests:	Krios1 ☐ Krios2 ☐ Talos ☐

Grid Box Name:

Grid Box Name:

Grid Box Name:

Grid Box Name:

FOR STAFF USE ONLY

Puck & Slot:	Puck & Slot:
Auto Grid Box Name:	Auto Grid Box Name:
1 Grid Box Position	1 Grid Box Position
2 Grid Box Position	2 Grid Box Position
3 Grid Box Position	3 Grid Box Position
4 Grid Box Position	4 Grid Box Position
Clipped By Date	Clipped By Date

Puck & Slot:	Puck & Slot:
Auto Grid Box Name:	Auto Grid Box Name:
1 Grid Box Position	1 Grid Box Position
2 Grid Box Position	2 Grid Box Position
3 Grid Box Position	3 Grid Box Position
4 Grid Box Position	4 Grid Box Position
Clipped By Date	Clipped By Date

Cab and cassette:

Circle cartridges to save

12	
11	
10	
9	
8	
7	
6	
5	
4	
3	
2	
1	

Box name

Car#

☐ Extra grids back in tube

☐ Grids from TEM back in tube

Rings		C-clips		Boxes	
Training		4TB HD		8TB HD	
